# UiO-66-NH_2_-Deposited Gold Nanoparticles Enable Enhanced Interference-Resistant Immunochromatographic Assay for Rapid Detection of Gentamicin in Animal-Derived Foods

**DOI:** 10.3390/foods14183264

**Published:** 2025-09-20

**Authors:** Yimeng Pang, Zehao Yang, Xiaohua Liu, Xing Shen, Hongtao Lei, Xiangmei Li

**Affiliations:** Guangdong Provincial Key Laboratory of Food Quality and Safety, College of Food Science, South China Agricultural University, Guangzhou 510642, China; pangyimeng928@stu.scau.edu.cn (Y.P.); yangzehao00@126.com (Z.Y.); liuxiaohua9903@163.com (X.L.); shenxing325@163.com (X.S.); hongtao@scau.edu.cn (H.L.)

**Keywords:** lateral flow immunoassay, metal-organic frameworks, gold nanoparticles, robust

## Abstract

Gentamicin (GEN) is a broad-spectrum antibiotic widely used in livestock production, and its excessive residues in animal-derived foods pose potential health risks to consumers. However, conventional colloidal gold immunochromatographic assays (AuNPs-ICA) often suffer from low sensitivity and poor tolerance to sample matrices. Herein, a UiO-66-NH_2_ framework decorated with gold nanoparticle (UiO-66-NH_2_@Au)-based ICA was employed to construct an ICA platform for GEN detection, combining the optical advantages of AuNPs with the protective and stable octahedral framework of the Metal-organic framework (MOF) to enhance antibody stability under extreme conditions. The method achieved limits of detection for GEN of 0.1 µg/kg in four tested matrices, with recoveries of 80.1–112.0% and coefficients of variation below 11.7%. Compared to traditional AuNPs-ICA, the sensitivity was improved by up to 30-fold, the pH tolerance range was expanded from 6–8 to 4–10, and the organic solvent tolerance to organic solvents expanded up to 40%. Verification with 40 real samples demonstrated excellent correlation (R^2^ > 0.99) with results from commercial ELISA kits. This UiO-66-NH_2_@Au-ICA platform offers a new technical solution with high sensitivity, strong good anti-interference performance, and robustness for rapid field detection of GEN residues in products of animal origin and holds significant practical importance for advancing rapid food safety detection technologies.

## 1. Introduction

Gentamicin (GEN) is a broad-spectrum aminoglycoside antibiotic that has been extensively used in veterinary medicine for the prevention and treatment of bacterial infections in products of animal origin [1,2]. However, excessive application can lead to measurable remnants in products of animal origin such as milk, pork, liver, and kidney [3]. Chronic exposure to GEN residues is associated with adverse health effects, including ototoxicity, nephrotoxicity, and the promotion of antimicrobial resistance [4,5]. Owing to such hazards, many jurisdictions have set stringent maximum residue thresholds (MRLs) for GEN in edible commodities, necessitating the creation of fast, highly sensitive, and dependable approaches for routine surveillance [6,7]. This has created an urgent need for swift, and precise analytical strategies that can be applied across multiple food matrices to support routine safety monitoring.

Conventional instrumental techniques, such as LC-MS/MS and HPLC [8], are widely regarded as reliable approaches for GEN analysis because of their high accuracy and sensitivity. Nevertheless, such approaches usually involve laborious sample preparation, costly equipment, and the need for skilled operators, which limits their application in field-based or large-scale screening scenarios. Immunochromatographic Assays (ICAs) have emerged as promising alternatives, offering simplicity, portability, and rapid results without the need for sophisticated equipment. Despite these advantages, traditional AuNP-based ICAs still face critical challenges, such as insufficient sensitivity to meet stringent regulatory requirements, limited tolerance to complex sample matrices, and reduced precision under variable environmental conditions [9,10].

Recent research has attempted to enhance ICA performance through the incorporation of novel nanomaterials and signal amplification strategies, such as enzyme-assisted catalysis, fluorescence labeling, and surface-enhanced Raman scattering [11]. While these approaches have improved assay sensitivity to some extent, most are hampered by narrow operational stability, reduced reproducibility in real samples, and susceptibility to interference from pH fluctuations or organic solvents [12,13]. Moreover, few studies have systematically addressed the balance between high sensitivity and robustness, which is crucial for practical application in diverse food matrices.

Metal-organic frameworks (MOFs) provide high porosity, large surface area, and tunable surface chemistry that are attractive for biomolecule loading and protection [14]. Multiple studies have shown that MOFs can shield protein and antibody (Ab) from thermal, chemical, and mechanical stress while allowing high loading, providing a rationale for MOF-based immunoprobes [15,16]. Among zirconium MOFs, UiO-66-NH_2_ is notable for chemical robustness and amino groups that facilitate covalent or electrostatic immobilization; UiO-66 frameworks are structurally stable in various solvents and under different pH, and UiO-66-NH_2_ has been used as a reliable platform for enzyme and Ab immobilization in sensing [17]. When Au nanoparticles are assembled onto UiO-66-NH_2_, UiO-66-NH_2_@Au materials are formed, exhibiting strong plasmonic properties with a rigid, dispersible scaffold that improves signal readout and operational stability versus conventional AuNP probes [18,19]. Moreover, recent MOF-based ICAs have primarily targeted hydrophobic or neutral analytes such as enrofloxacin, carbofuran, and sulfonylureas [17,20,21], whereas systematic studies on hydrophilic, polycationic aminoglycosides remain scarce. In addition, the robustness of MOF-based ICAs under variable conditions such as pH, ionic strength, and organic solvents has rarely been investigated.

In this study, UiO-66-NH_2_@Au nanocarriers together with conventional AuNPs were independently prepared to construct two types of immunochromatographic assays (ICAs) for the detection of GEN in milk, pork, liver, and kidney. A systematic assessment of sensitivity, stability, and robustness indicated that UiO-66-NH_2_@Au exhibited superior performance as an Ab carrier. This study provides an effective and broadly applicable strategy for developing rapid immunoassays with high sensitivity and stability, suitable for diverse complex food matrices.

## 2. Materials and Methods

### 2.1. Materials and Equipment

Gentamicin (GEN, 59.0%) was purchased from Shanghai McLean Biochemical Technology Co., Ltd. Anti-GEN antibody (23.5 mg/mL) and the coating antigen (GEN-OVA, 2.82 mg/mL) were prepared in-house. Goat anti-mouse IgG (17.5 mg/mL) and bovine serum albumin (BSA) were obtained from Sigma-Aldrich. Zirconium chloride (ZrCl_4_ ≥98%) and 2-aminoterephthalic acid (≥98%) were purchased from Macklin and Aladdin, respectively, and used as UiO-66-NH_2_ precursors. Nitrocellulose membranes (CN 95) were obtained from Sartorius AG (Unisart®, Göttingen, Germany). Sample pads (GF-2), absorbent pads (CH37K), and PVC backing cards were supplied by Shanghai Jinbiao Biotechnology Co., Ltd. An XYZ 3060 dispenser (BioDot, Irvine, CA, USA) and a ZQ 2000 strip cutter (Kinbio, Shanghai, China) were used for assembling the test strips. Transmission electron microscopy (FEI Talos L120C, FEI Company, Hillsboro, OR, USA), UV–Vis spectrophotometry (Thermo Fisher Scientific, Waltham, MA, USA), and dynamic light scattering (Nano ZS 90, Malvern, UK) were used for material characterization. A multifunctional strip reader (FIC-Q1, Hemai, China) was applied for signal acquisition.

### 2.2. Preparation of AuNPs and UiO-66-NH_2_@Au

The preparation procedures for AuNPs and UiO-66-NH_2_@Au followed the protocols established in our previous work [19], with detailed synthetic steps provided in the Supporting Information.

### 2.3. Preparation of UiO-66-NH_2_@Au-Ab Probe

**Scheme 1:**
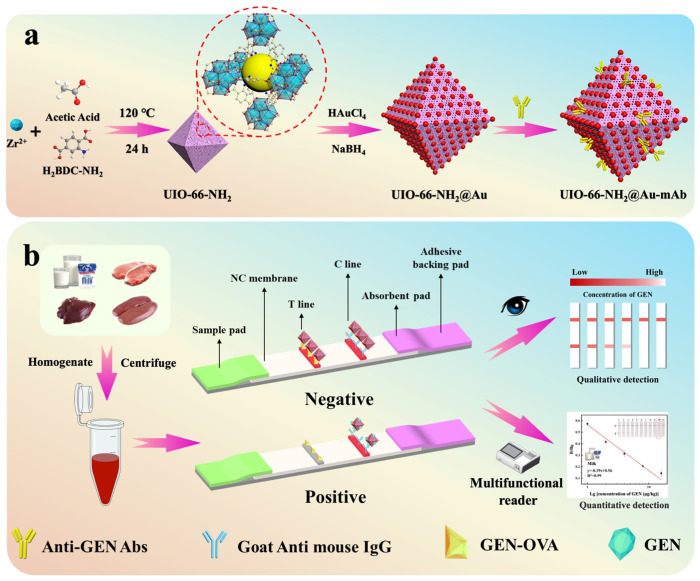
Schematic illustration of UiO-66-NH_2_@Au-based ICA for GEN detection. (**a**) Preparation of UiO-66-NH_2_@Au and conjugation with Ab to form probes. (**b**) Detection principle: in the competitive ICA format, GEN in the sample competes with GEN-OVA on the T line for probe binding. Higher GEN levels yield weaker T-line signals, while the C line serves as a control. T-line intensity is inversely correlated with GEN concentration and can be read visually or with a strip reader.

A 1% suspension of UiO-66-NH_2_@Au (1 mL) was adjusted to the desired pH by adding 6 μL of K_2_CO_3_ solution (0.02 M). Anti-GEN monoclonal Ab (23.5 μg) was added and incubated at room temperature for 20 min under gentle stirring. To block residual active sites, 40 μL of 20% BSA solution was added, followed by an additional incubation of 15 min. The reaction mixture was centrifuged at 10,000 rpm for 10 min at 4 °C, and the supernatant was discarded. The collected pellet (UiO-66-NH_2_@Au-Ab probe) was then resuspended in 200 μL phosphate buffer (0.02 M, pH 7.4) containing 5% sucrose, 0.3% PVP, 0.5% BSA, 0.2% Tween-20, and 0.03% ProClin-300 for storage,  as illustrated in Scheme 1a.

### 2.4. Sample Preparation

GEN-free milk and animal tissues (pork, liver, and kidney) were obtained from local supermarkets in Guangzhou (China) and confirmed to be free of GEN residues using commercial ELISA verification. Milk samples were analyzed directly without additional pretreatment. For tissue samples, 1 g was placed in a tube containing 2 mL of borate buffer (0.02 M, pH 8.0, 0.15 M NaCl)). The mixture underwent homogenization with a high-speed tissue grinder, followed by vortexing for 2 min. It was then subjected to centrifugation (4000 rpm, 10 min), and the resulting supernatant was collected as the test solution.

### 2.5. Assembly of ICA Strip

The ICA strips were prepared following the procedures described in our previous work [19], with technical details provided in Scheme 1b and Table S1.

### 2.6. Performance Evaluation of UiO-66-NH_2_@Au-ICA and AuNPs-ICA

#### 2.6.1. Sensitivity

Milk, pork, liver, and kidney samples were fortified with varying GEN concentrations to determine the cut-off values, calibration curves, limits of detection (LOD), and limits of quantification (LOQ). Spiking levels are expressed in μg/kg based on sample weight, with GEN standards added proportionally to the tissue mass. The cut-off value was defined as the minimum concentration of GEN at which the T-line disappeared under visual inspection. Calibration curves were generated by plotting log-transformed GEN concentrations (log10) on the *x*-axis against the B/B_0_ ratios on the *y*-axis. In this context, B/B_0_ referred to the ratio of T-line to C-line peak areas for fortified (B) and blank (B_0_) samples. The LOD and LOQ were estimated as the average response of 20 blank replicates plus three or ten times the standard deviation (SD) [22]. All spiking levels and blank controls were analyzed in triplicate (*n* = 3), and data are expressed as mean ± SD. $\bar{X}$ = B/B_0_ of 20 blank samples; σ = standard deviation.

$$LODs=\bar{X}+3\sigma$$

$$LOQs=\bar{X}+10\sigma$$

#### 2.6.2. The Selectivity, Accuracy, and Precision Evaluations

Details of the selectivity, accuracy, and precision evaluations are provided in the Supporting Information.

#### 2.6.3. Confirmatory Test

To further verify the reliability and practical applicability of the proposed method, a total of 40 GEN-free milk, pork, liver, and kidney samples (10 from each matrix) were purchased from local markets and confirmed to be negative by ELISA. Each sample was individually fortified with varying concentrations of GEN, and the spiking levels were blinded to the analyst performing the assays. All samples were tested in parallel using UiO-66-NH_2_@Au-ICA, conventional AuNPs-ICA, and a commercial ELISA kit. The detailed procedures for ELISA are provided in Table S2. Each spiked sample was analyzed in triplicate (*n* = 3), and results are reported as mean ± SD. Correlation analysis among those three methods was evaluated using linear regression (R^2^), and differences were assessed by t-test, with *p* < 0.05 considered significant.

##### Robustness Performance Analysis of UiO-66-NH_2_@Au-ICA and AuNPs-ICA

Deionized water was adjusted to pH values ranging from 1 to 14 using 1 M HCl or 1 M NaOH. A 150 μL aliquot of each pH-adjusted solution was transferred into a microwell, followed by the addition of UiO-66-NH_2_@Au-Ab or AuNPs-Ab probe, and the mixture was gently mixed to ensure uniform dispersion.

$$\mathrm{Inhibition}\;\mathrm{rate}\;(\%)=\frac{T/C_{\mathrm{blank}}-T/C_{\mathrm{sample}}}{T/C_{\mathrm{blank}}}\times 100$$

where T/C sample is the signal ratio for the tested condition and T/C blank is the signal ratio for the blank (no analyte). All inhibition rates were normalized to the optimal condition and expressed as a percentage. A normalized inhibition rate ≥ 70% was defined as “acceptable robustness.” Each condition was tested in triplicate (*n* = 3). The same procedure was applied for NaCl solutions with concentrations ranging from 0.005 M to 2 M and for methanol-water mixtures containing 10–80% (*v*/*v*) methanol. Salt and organic solvent tolerance was determined based on whether the inhibition rate met the robustness criterion.

## 3. Results

### 3.1. Characteristics of UiO-66-NH_2_, UiO-66-NH_2_@Au and UiO-66-NH_2_@Au-Ab

The characterization results of UiO-66-NH_2_, UiO-66-NH_2_@Au, and UiO-66-NH_2_@Au-Ab were consistent with our previous report [19]. The average particle size was 289 nm, providing suitable flow performance on ICA strips.

### 3.2. The Stability of the UiO-66-NH_2_@Au and AuNPs

**Figure 1:**
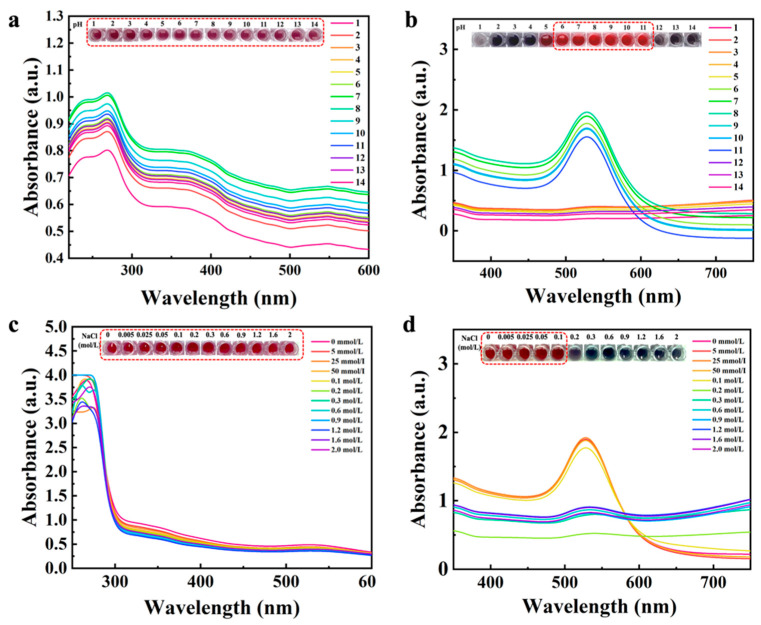
Effect of pH and ionic strength on the stability of UiO-66-NH_2_@Au and conventional AuNPs. (**a**) UV–vis absorption spectra of UiO-66-NH_2_@Au under different pH conditions (pH 2–14). (**b**) UV–vis absorption spectra of AuNPs under different pH conditions (pH 2–14). (**c**) UV–vis absorption spectra of AuNPs at various NaCl concentrations (0–2.0 mol/L). (**d**) UV–vis absorption spectra of UiO-66-NH_2_@Au at various NaCl concentrations (0–2.0 mol/L). Insets show the corresponding color changes of nanoparticle dispersions under the tested conditions.

To compare the environmental stability of UiO-66-NH_2_@Au and AuNPs, their UV-Vis absorption spectra were measured under different pH (1–14) and NaCl concentrations (0–2 M). In Figure 1a, UiO-66-NH_2_@Au showed clear and stable absorption peaks across the full pH range. This indicates that both acidic and alkaline conditions had little effect on its color and dispersion. This stability was attributed to the abundant -NH_2_ groups on the UiO-66-NH_2_ surface, which provide strong surface charge and help maintain colloidal dispersion in aqueous media. In contrast, AuNPs (Figure 1b) showed well-defined peaks only between pH 6–11, while significant aggregation and precipitation occurred under more acidic or alkaline conditions. This instability is consistent with the known sensitivity of citrate-stabilized AuNPs, in which the loss of surface charge leads to rapid aggregation. Consequently, AuNP-based ICAs may yield false negatives or poor reproducibility when applied to acidic beverages or alkaline animal tissue extracts. A similar trend was observed in salt tolerance tests. When the NaCl concentration exceeded 0.1 M, the absorption peak of AuNPs (Figure 1c) disappeared, and the colloid color changed from red to black, indicating rapid aggregation. By contrast, UiO-66-NH_2_@Au (Figure 1d) maintained both stable color and absorption peaks over the entire NaCl concentration range tested (0–2 M). This 20-fold higher salt tolerance highlights the strong ionic shielding and steric stabilization provided by the MOF scaffold. These effects prevent close contact of AuNPs even in highly ionic environments. The UiO-66-NH_2_@Au suspension also maintained good long-term stability during storage at 4 °C for six months, as shown in Figure S2.

### 3.3. Optimization of the UiO-66-NH_2_@Au-ICA

#### 3.3.1. The Coupling pH Value

**Figure 2:**
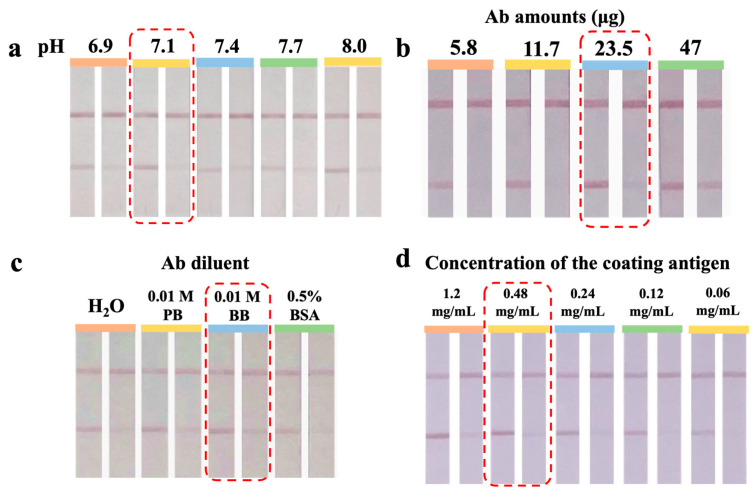
Optimization of UiO-66-NH_2_@Au-ICA. (**a**) Effect of coupling pH (6.9–8.0). The test strip showed the best color development and inhibition at pH 7.1. (**b**) Effect of Ab dosage (5.8–47 μg). Increasing the Ab amount improved T-line intensity up to 23.5 μg, but a further increase to 47 μg reduced inhibition efficiency. (**c**) Effect of Ab diluents. 0.01 M BB gave the clearest T-line and strongest inhibition compared with PB, water, or BSA solution. (**d**) Effect of coating antigen concentration (0.06–1.2 mg/mL). Strong signals were observed at high concentrations, but optimal balance of visibility and inhibition was achieved at 0.48 mg/mL. All experiments were repeated in triplicate (*n* = 3), and optimal conditions are highlighted with red dashed boxes.

Electrostatic adsorption governs the binding of Ab with UiO-66-NH_2_@Au. An appropriate pH can enhance the adsorption of basic amino acid residues on UiO-66-NH_2_@Au, thereby promoting Fab exposure and improving immunochromatographic sensitivity [23]. Figure 2a showed that the test strip exhibited optimal color development and inhibition at pH 7.1. Therefore, pH 7.1 was used as the optimal labeling condition. At lower pH values (<7.0), partial protonation of both the Ab and the -NH_2_ groups on UiO-66-NH_2_ likely weakened effective adsorption. This may have led to unfavorable Ab orientations and reduced binding efficiency. Conversely, at higher pH (>7.5), the degree of protonation decreased, reducing electrostatic interactions and thus lowering the conjugation yield. Thus, pH 7.1 was chosen as it balanced electrostatic attraction with preservation of Ab structural integrity.

#### 3.3.2. Optimization of Ab Amount

An insufficient amount of Ab may lead to weak or even absent color development on the T-line. Conversely, excessive Ab loading can result in Ab crowding on the UiO-66-NH_2_@Au surface, which may block coating antigen-binding sites, reduce sensitivity, and increase assay cost [24]. As shown in Figure 2b, increasing the Ab amount from 5.8 to 23.5 μg gradually increased T-line intensity and improved inhibition performance, suggesting that more available binding sites favored effective antigen–Ab recognition. However, a further increase to 47 μg led to a decrease in inhibition efficiency, indicating oversaturation of the particle surface and impaired competitive binding. Therefore, 23.5 μg was selected as the optimal Ab amount. This balance not only ensures strong specific signals but also avoids unnecessary consumption of antibodies, which are among the most expensive assay components.

#### 3.3.3. Optimization of Ab Diluent

The Ab diluent influences both Ab stability and its conjugation efficiency with UiO-66-NH_2_@Au [25]. As shown in Figure 2c, 0.01 M borate buffer (BB, pH 8.0) showed the best balance of T-line color development and inhibition compared with PB, water, or BSA solution. The slightly alkaline environment of BB likely maintained the tertiary structure of the Ab, preserved the activity of its antigen-binding sites, and enhanced electrostatic interactions with the amino-functionalized MOF surface. In contrast, water lacked buffering capacity and led to reduced reproducibility, while the presence of high concentrations of BSA might have competitively blocked active sites, resulting in weaker binding. Therefore, 0.01 M BB was selected as the optimal diluent.

#### 3.3.4. Optimization of the Concentration of the Coating Antigen

The concentration of coating antigen determines the visual intensity of the T-line and the competitive binding dynamics [20]. As shown in Figure 2d, progressive dilution of the coating antigen from 1.2 to 0.06 mg/mL resulted in a corresponding decrease in T-line intensity. At high antigen concentrations, the T-line signal was strong but competition with free gentamicin was insufficient, thereby lowering sensitivity. Conversely, at very low coating concentrations, the weak T-line reduced visual clarity and compromised assay reliability. Taking both color development and inhibition efficiency into account, 0.48 mg/mL was selected as the optimal coating antigen concentration. This condition provides a balance between maintaining a clear and easily distinguishable T-line and ensuring sufficient competitive binding.

### 3.4. Evaluation of UiO-66-NH_2_@Au-ICA and AuNPs-ICA

#### 3.4.1. Analytical Sensitivity

**Figure 3:**
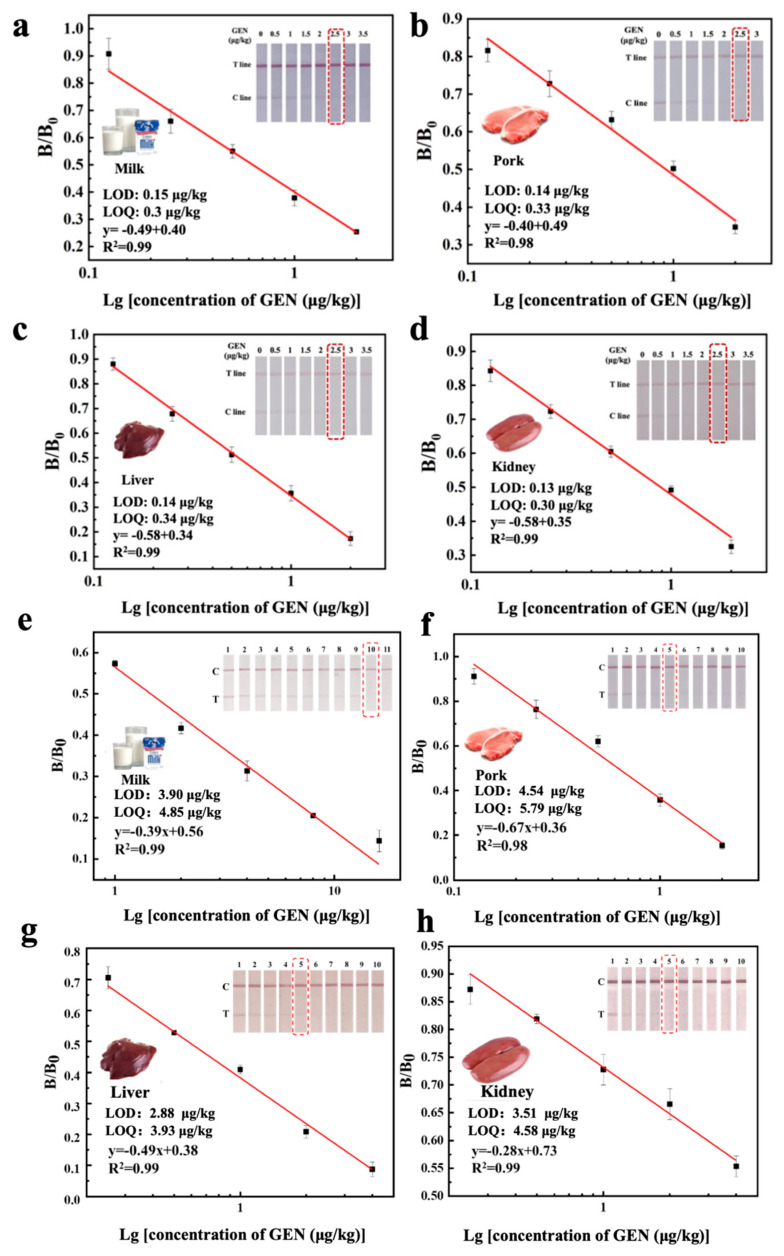
The cut-off values and standard curves of GEN in (**a**) milk, (**b**) pork, (**c**) liver and (**d**) kidney samples of UiO-66-NH_2_@Au-ICA. The cut-off values and standard curves of GEN in (**e**) milk, (**f**) pork, (**g**) liver and (**h**) kidney samples of AuNPs-ICA. Each inset shows photographs of ICA test strips tested with GEN-spiked samples at representative concentrations. The cut-off value was defined as the lowest concentration at which the test line (T) completely disappeared.

As shown in Figure 3, the cut-off values of UiO-66-NH_2_@Au-ICA for GEN in milk, pork, liver, and kidney were 2.5 μg/kg. The LODs were 0.15, 0.10, 0.14, and 0.13 μg/kg, while the LOQs were 0.30, 0.33, 0.34, and 0.30 μg/kg. The detection ranges extended from 0.12 to 12.0 μg/kg. In comparison, the cut-off values of the AuNPs-ICA were 10, 5, 5, and 5 μg/kg for milk, pork, liver, and kidney, with LODs of 3.90, 4.54, 4.88, and 3.51 μg/kg, and LOQs of 4.85, 5.79, 3.93, and 4.58 μg/kg, respectively. The corresponding linear ranges were 1.0–4.0 μg/kg. Therefore, the UiO-66-NH_2_@Au-ICA exhibited up to a 32-fold improvement in sensitivity compared with the conventional AuNPs-ICA.

#### 3.4.2. Cross-Reactivity Analysis

**Figure 4:**
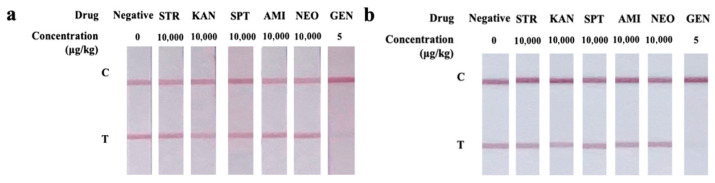
Selectivity analysis of (**a**) UiO-66-NH_2_@Au-ICA and (**b**) AuNPs-ICA. UiO-66-NH_2_@Au-ICA and AuNPs-ICA were tested with STR, KAN, SPT, AMI, and NEO, each tested at 10,000 µg/kg, while GEN was spiked at 5 µg/kg as the target analyte. Negative samples contained no target.

Figure 4 and Table 1 summarize the selectivity findings. Anti-GEN Ab exhibited no CR with other aminoglycoside antibiotics (STR, KAN, SPT, AMI, NEO), due to the distinct structural differences between these compounds and GEN. Consistent selectivity patterns were observed for UiO-66-NH_2_@Au-ICA, AuNPs-ICA, and icELISA.

#### 3.4.3. Accuracy and Precision

For UiO-66-NH_2_@Au-ICA, the recovery rates for GEN detection were 89.6–112.0% in milk, 95.8–110.7% in pork, 93.6–109.7% in liver, and 80.1–106.1% in kidney. The corresponding CVs ranged from 6.8–11.7%, 4.5–5.8%, 1.3–8.2%, and 4.0–5.5%. In comparison, the recovery rates of AuNPs-ICA in milk, pork, liver, and kidney were 81.7–113.5%, 81.4–87.4%, 82.1–118.3%, and 82.4–97.8%, respectively, with CVs ranging from 5.6–14.8%, 2.0–8.8%, 1.8–12.9%, and 2.3–6.4%, respectively (Table 2). These results demonstrated that both UiO-66-NH_2_@Au-ICA and AuNPs-ICA provided good accuracy and reproducibility.

#### 3.4.4. Confirmatory Test Results

The results of the confirmatory tests are summarized in Table 3 and illustrated in Figure S1. Linear regression analysis showed strong correlations between the ICA methods and the commercial ELISA, with R^2^ values of 0.99 for both UiO-66-NH_2_@Au-ICA and AuNPs-ICA (Figure S1a,c). The analysis of 40 blind spiked samples across four matrices further confirmed the close agreement between ICA methods and ELISA (Figure S1b,d). To provide stronger evidence of consistency, Bland–Altman analysis was performed (Figure S1d,e). UiO-66-NH_2_@Au-ICA showed a mean bias of only 12.7 µg/kg and narrow 95% limits of agreement (−131.4 to 156.8 µg/kg), indicating strong agreement with ELISA across the tested concentration range. In comparison, AuNPs-ICA also showed acceptable agreement but with slightly larger deviations at higher concentrations. These results demonstrate that the UiO-66-NH_2_@Au-ICA method is accurate, robust, and suitable for practical monitoring of GEN residues in milk, pork, liver, and kidney samples.

##### 3.5. Robustness Characteristics of UiO-66-NH_2_@Au-ICA Compared with AuNPs-ICA

**Figure 5:**
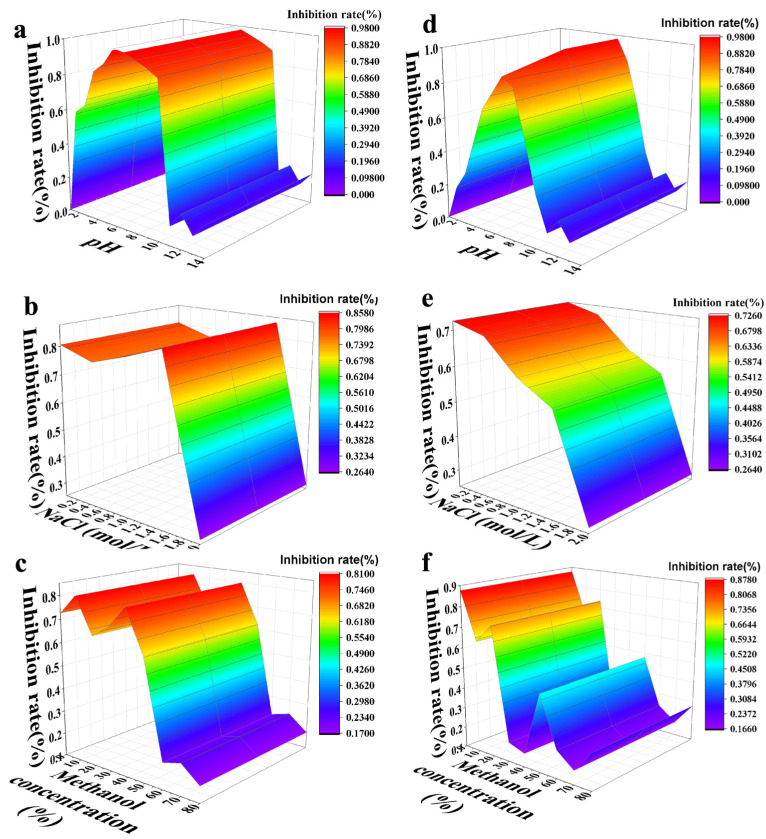
Robustness comparison of UiO-66-NH_2_@Au-ICA (**a**–**c**) and AuNPs-ICA (**d**–**f**) under different assay conditions. (**a**,**d**) Effect of pH values (3.0–12.0). UiO-66-NH_2_@Au-ICA maintained inhibition rates above 70% over pH 4–10, whereas AuNPs-ICA was stable only in the narrow range of pH 6–8. (**b**,**e**) Effect of NaCl concentrations (0–1.2 M). UiO-66-NH_2_@Au-ICA remained stable up to 1.2 M NaCl, while AuNPs-ICA dropped below the 70% threshold at 0.3 M. (**c**,**f**) Effect of methanol concentrations (0–40%, *v*/*v*). UiO-66-NH_2_@Au-ICA preserved acceptable inhibition rates up to 40% methanol, compared with only 20% for AuNPs-ICA. Each condition was tested in triplicate (*n* = 3). Inhibition rates were normalized to optimal conditions (pH 7.4, 0 M NaCl, 0% MeOH), and ≥70% was considered acceptable robustness.

Figure 5a showed that UiO-66-NH_2_@Au-ICA maintained inhibition rates above 70% over a broad pH range of 4–10, indicating stable performance from mildly acidic to strongly alkaline conditions. In contrast, AuNPs-ICA (Figure 5d) exhibited acceptable performance only within a narrow pH range of 6–8, with a rapid decline under more extreme conditions. When exposed to increasing NaCl concentrations, UiO-66-NH_2_@Au-ICA maintained inhibition rates ≥70% up to 1.2 M NaCl (Figure 5b). In contrast, AuNPs-ICA performance fell below the threshold at 0.3 M (Figure 5e), demonstrating a 4-fold improvement in salt tolerance. Similarly, UiO-66-NH_2_@Au-ICA maintained inhibition rates in methanol-water mixtures containing up to 40% (*v*/*v*) methanol, compared with only 20% for AuNPs-ICA (Figure 5c,f), representing a 2-fold improvement in organic solvent tolerance. The superior stability was attributed to the larger particle size and rigid octahedral framework of UiO-66-NH_2_@Au. This structure prevents aggregation and shields Ab binding sites under harsh conditions, thereby preserving both antigen-binding capability and signal generation performance. In addition, the abundant –NH_2_ groups on UiO-66-NH_2_ can be protonated in aqueous media, which provides electrostatic stabilization against particle aggregation [14]. These amino functionalities also facilitate hydrogen bonding and electrostatic interactions with antibodies, thereby maintaining their native conformation and activity under extreme pH, high ionic strength, or organic solvent conditions [21]. Thus, these features explain the significantly improved robustness of UiO-66-NH_2_@Au compared with conventional AuNPs.

##### 3.6. Comparison of ICAs for GEN Detection

ICAs for detecting GEN are summarized in Table 4. (1) In contrast to conventional methods that demand multiple pretreatment procedures, this approach enables direct detection of GEN in milk and requires only simple extraction and centrifugation for pork, liver, and kidney, the main sites of GEN residue accumulation. (2) The method is applicable to a wider range of animal-derived foods, ensuring broad adaptability across matrices. (3) The UiO-66-NH_2_@Au-ICA demonstrated tolerance ranges 2.3–4.0 times broader toward pH, ionic strength, and organic solvents compared with AuNPs-ICA. This improvement is attributed to the larger particle size and rigid octahedral framework of UiO-66-NH_2_@Au that provided enhanced structural stability. (4) In terms of analytical performance, the sensitivity of UiO-66-NH_2_@Au-ICA was improved by 2–32 times relative to conventional AuNPs-ICA. The established UiO-66-NH_2_@Au-ICA combined simple pretreatment, broad sample compatibility, high robustness, and superior sensitivity, making it a reliable and practical tool for GEN detection in products of animal origin.

Despite these promising results, some limitations remain. The present study has not yet been validated under large-scale manufacturing or field-use conditions, which will be essential for future commercialization. In addition, while the assay was successfully applied to milk, pork, liver, and kidney, its applicability to complex processed foods (e.g., high-fat or fermented products) still requires further evaluation. Such matrices often contain high levels of lipids, proteins, or fermentation by-products that may interfere with antibody–antigen interactions, making further validation essential to demonstrate robustness and reliability in real-world applications. Future work will focus on field validation, large-scale production assessment, and integration with digital readout systems to further improve quantitative accuracy.

## 4. Conclusions

In this study, a robust and highly sensitive UiO-66-NH_2_@Au-ICA was developed for the rapid detection of gentamicin residues in milk, pork, liver, and kidney. The assay used the stable octahedral UiO-66-NH_2_ structure as an Ab carrier while retaining the optical properties of AuNPs. It achieved an LOD of 0.1 µg/kg in all tested matrices, representing a 2–32-fold improvement in sensitivity over AuNPs-ICA. The UiO-66-NH_2_@Au-ICA exhibited a broadened pH tolerance range (4–10) and doubled resistance to organic solvents (up to 40%), ensuring stable performance in complex food matrices. The results of testing 40 real samples were found to be basically consistent with those obtained by commercial (ELISA) kits. These results indicate that the method is practical and provides a sensitive, rapid, and robust strategy for GEN monitoring in animal products.

## Figures and Tables

**Table 1:** The IC_50_ and CR values of anti-GEN Ab determined by icELISA, UiO-66-NH_2_@Au-ICA and AuNPs-ICA.

Analyst	icELISA		UiO-66-NH_2_@Au-ICA		AuNPs-ICA	
	IC_50_ (μg/kg)	CR%	IC_50_ (μg/kg)	CR%	IC_50_ (μg/kg)	CR%
Gentamicin	3.26	100	2.44	100	2.85	100
Streptomycin	>10,000	<0.1	>10,000	<0.1	>10,000	<0.1
Kanamycin	>10,000	<0.1	>10,000	<0.1	>10,000	<0.1
Spectinomycin	>10,000	<0.1	>10,000	<0.1	>10,000	<0.1
Amikacin	>10,000	<0.1	>10,000	<0.1	>10,000	<0.1
Neomycin	>10,000	<0.1	>10,000	<0.1	>10,000	<0.1

**Table 2:** Recovery of the UiO-66-NH_2_@Au-ICA for the determination of GEN in milk, pork, liver and kidney (*n* = 3).

Sample	UiO-66-NH_2_@Au-ICA				AuNPs-ICA			
	Spiked Level (μg/kg)	Measured Level (μg/kg)	Recovery (%)	CV (%)	Spiked Level (μg/kg)	Measured Level (μg/kg)	Recovery (%)	CV (%)
milk	0.20	0.22 ± 0.03	112.0	11.70	1.50	1.88 ± 0.02	81.70	5.60
	0.60	0.56 ± 0.02	93.80	6.80	7.50	9.01 ± 0.05	85.20	14.10
	1.50	1.34 ± 0.02	89.60	9.30	15.0	13.39 ± 0.05	113.50	14.80
pork	0.20	0.21 ± 0.01	107.30	5.10	0.20	0.23 ± 0.03	87.40	2.80
	0.60	0.66 ± 0.04	110.70	5.80	0.60	0.73 ± 0.04	81.40	2.00
	1.50	1.43 ± 0.05	95.80	4.50	1.50	1.76 ± 0.12	85.20	8.80
liver	0.20	0.22 ± 0.07	109.70	1.30	0.30	0.37 ± 0.01	82.10	1.80
	0.60	0.56 ± 0.04	93.60	2.50	1.50	1.69 ± 0.05	88.80	4.70
	1.50	1.63 ± 0.05	108.70	8.20	3.0	2.55 ± 0.09	118.30	12.90
kidney	0.20	0.16 ± 0.11	80.10	5.50	0.30	0.32 ± 0.02	97.80	3.50
	0.60	0.63 ± 0.03	106.10	4.40	1.50	1.58 ± 0.06	96.80	2.30
	1.50	1.58 ± 0.02	105.60	4.00	3.00	3.67 ± 0.12	82.40	6.40

Values are mean ± SD (*n* = 3). Statistical significance was evaluated by *t*-test.

**Table 3:** The determination of GEN in milk, pork, liver and kidney by commercial ELISA kit, UIO-66-NH_2_@Au-ICA, and AuNPs-ICA (*n* = 3).

Sample	Commercial ELISA Kit		UiO-66-NH_2_@Au-ICA		AuNPs-ICA	
	Mean ± SD	CV (%)	Mean ± SD	CV (%)	Mean ± SD	CV (%)
	(μg/kg)		(μg/kg)		**(μg/kg)**	
**Milk (** ***n*** **= 10)**						
Milk 1	ND	-	ND	-	ND	-
Milk 2	ND	-	ND	-	ND	-
Milk 3	ND	-	ND	-	ND	-
Milk 4	2.34 ± 0.32	13.68%	2.13 ± 0.25	11.74%	2.12 ± 0.13	6.13%
Milk 5	2.59 ± 0.39	15.06%	2.71 ± 0.32	11.81%	2.71 ± 0.29	10.70%
Milk 6	5.53 ± 0.66	11.93%	5.77 ± 0.28	4.85%	5.27 ± 0.69	13.09%
Milk 7	5.76 ± 0.55	9.55%	4.97 ± 0.31	6.24%	4.99 ± 0.73	14.63%
Milk 8	10.91 ± 0.71	6.51%	9.87 ± 0.34	3.44%	9.57 ± 0.57	5.96%
Milk 9	99.6 ± 3.71	3.72%	98.86 ± 3.34	3.38%	100.45 ± 3.88	3.86%
Milk 10	95.98 ± 5.13	5.34%	103.99 ± 4.12	3.96%	99.4 ± 2.57	2.59%
**Pork (** ***n*** **= 10)**						
Pork 1	ND	-	ND	-	ND	-
Pork 2	ND	-	ND	-	ND	-
Pork 3	ND	-	ND	-	ND	-
Pork 4	2.42 ± 0.08	3.31%	2.56 ± 0.35	13.67%	2.66 ± 0.35	13.16%
Pork 5	2.76 ± 0.39	14.13%	2.44 ± 0.27	11.07%	2.68 ± 0.19	7.09%
Pork 6	5.82 ± 0.99	17.01%	6.16 ± 0.87	14.12%	6.12 ± 0.86	14.05%
Pork 7	5.45 ± 0.33	6.06%	5.78 ± 0.45	7.79%	4.98 ± 0.44	8.84%
Pork 8	10.24 ± 0.51	4.98%	10.12 ± 0.98	9.68%	10.22 ± 1.01	9.88%
Pork 9	50.8 ± 4.89	9.63%	48.67 ± 4.89	10.05%	49.77 ± 5.71	11.47%
Pork 10	101.22 ± 6.65	6.57%	100.55 ± 7.18	7.14%	104.24 ± 7.73	7.42%
**Liver (** ***n*** **= 10)**						
Liver 1	ND	-	ND	-	ND	-
Liver 2	ND	-	ND	-	ND	-
Liver 3	5.32 ± 0.54	10.15%	4.87 ± 0.65	13.35%	6.01 ± 0.57	9.48%
Liver 4	5.19 ± 0.58	11.18%	4.91 ± 0.24	4.89%	4.94 ± 0.38	7.69%
Liver 5	10.16 ± 1.24	12.20%	9.22 ± 0.45	4.88%	12.05 ± 0.27	2.24%
Liver 6	10.02 ± 1.05	10.48%	9.9 ± 0.95	9.60%	9.99 ± 1.17	11.71%
Liver 7	99.07 ± 4.19	4.23%	108.77 ± 10.81	9.94%	105.43 ± 8.31	7.88%
Liver 8	105.19 ± 7.80	7.42%	96.24 ± 4.73	4.91%	110.96 ± 7.92	7.14%
Liver 9	2543.82 ± 208.23	8.19%	2671.33 ± 180.56	6.76%	2638.77 ± 199.48	7.56%
Liver 10	5051.11 ± 380.11	7.53%	5489.12 ± 550.81	10.03%	4985.9 ± 401.39	8.05%
**Kidney (** ***n*** **= 10)**						
Kidney 1	ND	-	ND	-	ND	-
Kidney 2	ND	-	ND	-	ND	-
Kidney 3	5.2 ± 0.12	2.31%	5.51 ± 0.37	6.72%	5.12 ± 0.48	9.38%
Kidney 4	5.24 ± 0.32	6.11%	4.92 ± 0.61	12.40%	4.69 ± 0.67	14.29%
Kidney 5	9.11 ± 0.78	8.56%	9.89 ± 1.23	12.44%	10.05 ± 1.35	13.43%
Kidney 6	9.13 ± 0.73	8.00%	11.7 ± 1.12	9.57%	9.35 ± 1.31	14.01%
Kidney 7	101.95 ± 7.46	7.32%	103.45 ± 12.01	11.61%	100.26 ± 8.71	8.69%
Kidney 8	99.21 ± 6.79	6.84%	101.11 ± 9.33	9.23%	105.37 ± 7.38	7.00%
Kidney 9	1030.12 ± 109.90	10.67%	1003.45 ± 120.92	12.05%	980.76 ± 104.88	10.69%
Kidney 10	2070.65 ± 170.67	8.24%	2019.12 ± 183.45	9.09%	1994.35 ± 150.46	7.54%

**Table 4:** Comparison of ICAs for GEN detection.

Method	Sample	Pretreatment	Assay Time	Cut-Off Value (μg/kg)	LOD	Robustness	Reference
			(min)		(μg/kg)		
AuNPs-ICA	Milk	Centrifuge after organic extraction	15	139.0	-	-	[26]
AuNPs-ICA	Milk	Dilution	10	100.0	-	-	[27]
AuNPs-ICA	Milk	Direct detection	-	20.0	-	-	[28]
AuNPs-ICA	Milk	-	-	20.0	-	-	[29]
QDs-ICA	Milk	Dilution	-	10.0	5.0	-	[30]
CG@PDA	Milk	Direct detection	8	5.0	1.51	-	[4]
	pork	centrifugation (pork, liver, kidney)		5.0	1.49	-	
	liver			5.0	1.61	-	
	kidney			5.0	1.75	-	
AuNPs-ICA	Milk	Direct detection centrifugation	8	10.0	3.90	pH range: 6 to 8 NaCl concentration (0–0.3 M) Methanol content: 0–20% (*v*/*v*)	Present study
	pork			5.0	4.54		
	liver			5.0	2.88		
	kidney			5.0	3.51		
UIO-66-NH_2_@Au-ICA	Milk	Direct detection (milk); centrifugation (pork, liver, kidney)	8	2.50	0.15	pH range: 4 to 10 NaCl concentration: (0–1.2 M) Methanol content: 0–40% (*v*/*v*)	Present study
	pork			2.50	0.14		
	liver			2.50	0.14		
	kidney			2.50	0.13		

Abbreviations: ICA, immunochromatographic assay; AuNPs, gold nanoparticles; QDs, quantum dots; CG@PDA, colloidal gold@polydopamine; UiO-66-NH_2_@Au, UiO-66-NH_2_-deposited gold nanoparticles; GEN.

## Data Availability

The original contributions presented in the study are included in the article/Supplementary Materials, further inquiries can be directed to the corresponding author.

## Supplementary Materials

The following supporting information can be downloaded at: https://www.mdpi.com/article/10.3390/foods14183264/s1, Figure S1: Comparison of UiO-66-NH_2_@Au-ICA and AuNPs-ICA with commercial ELISA for 40 spiked samples. (a,c) Linear regression analysis (R^2^ = 0.99). (b,d) Blind sample results across four matrices. (e,f) Bland–Altman plots showing good agreement with ELISA. UiO-66-NH_2_@Au-ICA: Bias = 12.7 µg/kg, 95% LOA = −131.4 to 156.8 µg/kg. AuNPs-ICA: Bias = −1.9 µg/kg, 95% LOA = −48.5 to 44.8 µg/kg. Figure S2: Stability of UiO-66-NH_2_@Au suspension during storage. The suspension was stored at 4 °C, and its absorbance at the characteristic plasmon peak was monitored monthly for six months (0–6 months, shown in inset photographs). Only slight fluctuations were observed, with no significant decline, confirming that the suspension maintained colloidal stability and was suitable for subsequent Ab conjugation and ICA preparation. Table S1: Working conditions of the developed UiO-66-NH_2_@Au-ICA. Table S2: Sample preparation procedures for the commercial ELISA kit.
