# Supplementary material for: UiO-66-NH2-Deposited Gold Nanoparticles Enable Enhanced Interference-Resistant Immunochromatographic Assay for Rapid Detection of Gentamicin in Animal-Derived Foods
Source: Foods. 2025 Sep 20;14(18):3264. doi: 10.3390/foods14183264 (PMC12469628; doi:10.3390/foods14183264)
Supplement: Supplementary file 1 [file foods-14-03264-s001.zip › foods-3863366-supplementary.pdf]

Support Information

UiO-66-NH_2-deposited gold nanoparticles enable enhanced interference-resistant immunochromatographic assay for rapid detection of gentamicin in animal-derived foods

Yimeng Pang, Zehao Yang, Xiaohua Liu, Xing Shen, Hongtao Lei, Xiangmei Li *

Guangdong Provincial Key Laboratory of Food Quality and Safety, College of Food Science, South China Agricultural University, Guangzhou 510642, China

* Corresponding author. Phone: +86 20 8528 3925. Fax: +86 20 8528 0270. E-mail: lixiangmei12@163.com

1. Synthesis of AuNPs and UiO-66-NH_2@Au

UiO-66-NH_2 was prepared by dissolving 2-aminoterephthalic acid (0.181 g) and $ZrCl_4$ (0.233 g) in 30 mL of DMF under constant stirring. Acetic acid (3.3 mL) was then introduced as a modulator, and the mixture was sealed and heated at 120 °C for 24 h. The resulting precipitate was recovered by centrifugation (10,000 rpm, 5 min), rinsed three times with fresh DMF to remove unreacted species, and subsequently dried at 100 °C for 12 h to obtain UiO-66-NH_2 crystals.

To synthesize UiO-66-NH_2@Au, $HAuCl_4 \cdot 4H_2O$ (40 μg, 1%) and UiO-66-NH_2 (0.5 mg) were dispersed in 5 mL deionized water and stirred until the suspension appeared light yellow. Then, 20 μL of freshly prepared $NaBH_4$ solution (1 M) was added, and the mixture was allowed to react for 10 min. The color gradually changed to red, confirming the successful deposition of Au nanoparticles onto the UiO-66-NH_2 framework.

2. Selectivity

The selectivity of the UiO-66-NH2@Au-ICA and AuNPs-ICA were assessed by detecting several common aminoglycoside antibiotics, including streptomycin (STR), kanamycin (KAN), spectinomycin (SPT), amikacin (AMI), and neomycin (NEO). Cross-reactions (CR, %) were performed by the UIO-66-NH_2@Au-ICA and the indirect competitive enzyme-linked immunosorbent assay (icELISA), respectively. The CR rates were obtained via the following equation:

$$\text{CR}\ (\%) = \frac{IC_{50\ (target)}}{IC_{50\ (analogue)}} \times 100\%$$

3. Accuracy and precision

Milk, pork, liver, and kidney samples confirmed to be GEN-free by a commercial ELISA kit were spiked with GEN standard solutions at three concentration levels. Sample pretreatment was carried out as described above. Each concentration level was

analyzed in quintuplicate on three separate days. The accuracy is expressed as the recovery rate, while the precision was evaluated by calculating the coefficient of variation (CV).

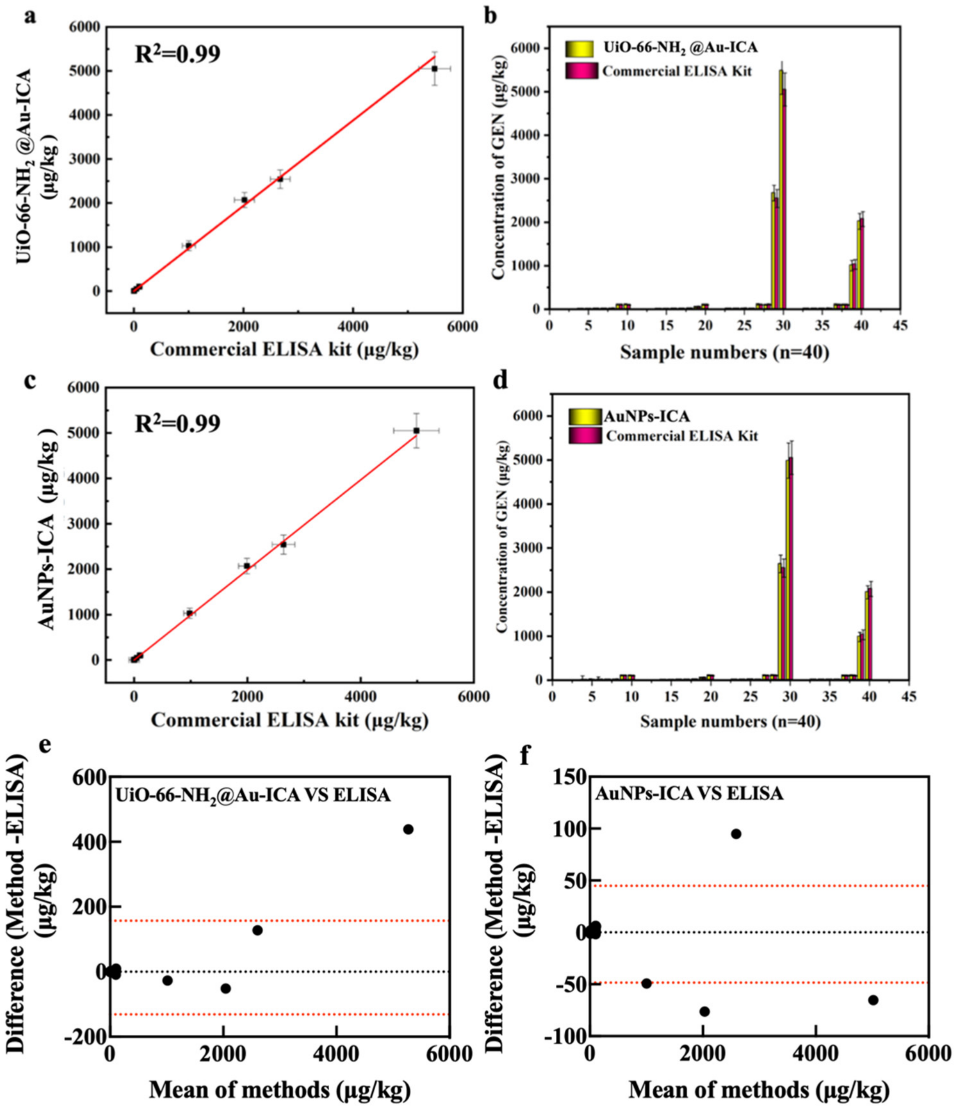

Figure S1. Comparison of UiO-66-NH_2@Au-ICA and AuNPs-ICA with commercial ELISA for 40 spiked samples. (a, c) Linear regression analysis (R^2 = 0.99). (b, d) Blind sample results across four matrices. (e, f) Bland–Altman plots showing good agreement with ELISA. UiO-66-NH_2@Au-ICA: Bias = 12.7 μg/kg, 95% LOA =

−131.4 to 156.8 μg/kg. AuNPs-ICA: Bias = −1.9 μg/kg, 95% LOA = −48.5 to 44.8 μg/kg.

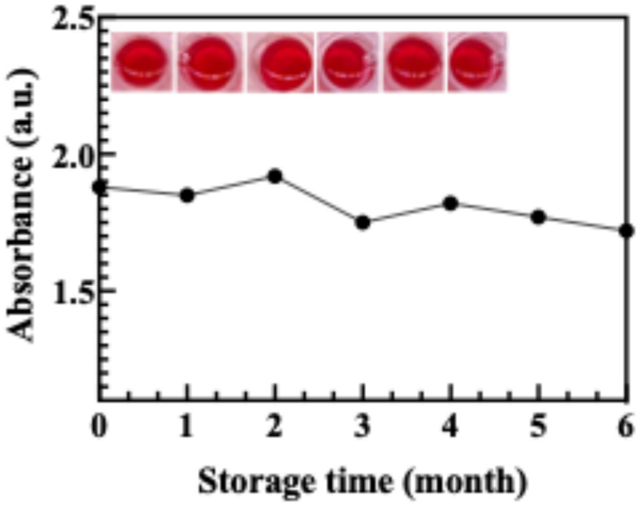

Figure S2. Stability of UiO-66-NH_2@Au suspension during storage. The suspension was stored at 4 °C, and its absorbance at the characteristic plasmon peak was monitored monthly for six months (0–6 months, shown in inset photographs). Only slight fluctuations were observed, with no significant decline, confirming that the suspension maintained colloidal stability and was suitable for subsequent Ab conjugation and ICA preparation.

Table S1 Working conditions of the developed UiO-66-NH_2@Au-ICA

Item	Working conditions
Control line	Goat anti-mouse IgG, 0.35 mg/mL, 0.8 μL/cm
Test line	GEN-OVA, 0.48 mg/mL, 0.8 μL/cm
Coating buffer for C line	0.02 M PBS (pH 7.4)
Coating buffer for T line	0.02 M PBS (pH 7.4)
Nitrocellulose membrane	UniSart CN 95
The mAb amount	23.5 μg
Blocking agent	20% BSA
Probe suspension	PB (0.02 M, pH 7.4, containing 5% sucrose, 0.3% PVP, 0.5% BSA, 0.2% tween-20, 0.03% procline-300)
Sample extract	0.02 M BBS (pH 8.0, 0.15 M NaCl)
Sample pad material	GF-2
Sample pad treatment formula	0.02 M PB (0.5% BSA, 1% PVP, 0.5% tween-20)
Sample preparation	Milk: detected directly without treatment; pork, liver, and kidney: 1 g sample+2 mL of sample extract

Table S2 Sample preparation procedures for the commercial ELISA kit.

Sample type	Step	Operation
**Milk**	1	Weigh 1 g of sample into a 5 mL centrifuge tube.
	2	Add 1 mL of 1% trichloroacetic acid and mix well for 1 min.
	3	Centrifuge at 5000×g for 5 min.
	4	Transfer 50 μL of supernatant into a 2 mL centrifuge tube.
	5	Add 950 μL of sample diluent, mix for 30 s, and use 50 μL of the prepared solution for testing.
**Animal tissue**	1	Add 10 g of homogenized sample into a 50 mL centrifuge tube.
	2	Add 10 mL of tissue extract into the tube and mix thoroughly for 1 min.
	3	Centrifuge at 5000×g for 5 min, and use 50 μL of supernatant for testing.
